# Identification of Characteristic Flavor Compounds and Quality Evaluation of *Butyriboletus roseoflavus* from Different Regions in Yunnan

**DOI:** 10.3390/foods14101676

**Published:** 2025-05-09

**Authors:** Ling Song, Qiming Zhao, Xuebin Shao, Xiangqian Lv, Juan Lu, Ruiping Luo, Yurong Liu, Xing Zhou, Qiang Li, Mingying Gui

**Affiliations:** 1 College of Food Science and Technology, Yunnan Agricultural University, Kunming 650201, China; 2 College of Agriculture and Biotechnology, Yunnan Agricultural University, Kunming 650201, China; 3 The State-Owned Forest Farm in Longyang District Baoshan City, Baoshan 678000, China; 4 Library, Yunnan Agricultural University, Kunming 650201, China; 5 Yunnan Plateau Characteristic Agricultural Industry Research Institute, Yunnan Agricultural University, Kunming 650201, China; 6 School of Food and Biological Engineering, Chengdu University, Chengdu 610106, China

**Keywords:** edible mushroom, aroma, taste, quality, geographic origin

## Abstract

*Butyriboletus roseoflavus* is a rare wild edible mushroom. Yet, the relationship between its chemical composition and quality, as well as the influence of geographic origin on its flavor profile, remains unclear. In this study, ultra-performance liquid chromatography–tandem mass spectrometry (UPLC-MS/MS) and headspace solid-phase microextraction coupled with gas chromatography–mass spectrometry (HS-SPME-GC-MS) were used to investigate flavor differences and influencing factors among samples from different regions. Seventeen key volatile compounds (OAV > 1) were identified, with α-pinene, styrene, octanal, 1,3,5-trithiane, and 2,4-undecadienal being the primary aroma contributors. Six characteristic taste-active compounds (TAV > 1) were detected, among which Glu, Ala, and His played dominant roles. Differential metabolites were mainly enriched in nucleotides and their derivatives, suggesting their importance in environmental adaptation and quality formation. Correlation analysis revealed that the abundance of key metabolites was closely related to geographic origin: temperature, humidity, light intensity, and CO_2_ concentration mainly influenced aroma variation, while taste differences were associated with soil electrical conductivity and microclimatic changes mediated by altitude. These findings provide a comprehensive understanding of the flavor characteristics of *B. roseoflavus* and offer a theoretical basis for its future processing and utilization.

## 1. Introduction

Yunnan Province, located in the southwestern border of China, features an inland low-latitude region with a plateau and mountainous terrain, and a subtropical monsoon climate. These unique geographical conditions create ideal growing conditions for wild mushrooms, while also offering abundant resources for wild mushroom research. Currently, 882 species of wild mushrooms have been identified in Yunnan, accounting for 91% of the national total [1]. The boletes in Yunnan are highly diverse [2], exhibiting distinct characteristics in terms of morphology, color, and flavor. Boletes are rich in protein, carbohydrates, and minerals, with a low lipid content. Their nutritional value is comparable to meat, eggs, and milk, aligning with contemporary trends toward natural, healthy diets. In addition, the potential medicinal value of Boletus species is considerable, including the enhancement of the immune system, antioxidant effects, and anti-tumor properties [3,4,5]. However, research on these edible wild boletes remains scarce, and many bolete species have yet to be identified. *B. roseoflavus*, a wild edible mushroom from the Boletaceae family and the Boletus genus, is known for its distinct coloration. Its cap ranges from light pink to rose, the stem is yellow to lemon-yellow with a red net-like pattern, and the fruiting body turns blue when damaged. Due to its unique aroma and taste, it is highly favored by consumers. Research on *B. roseoflavus* has been limited, primarily focusing on its biological activity and ecology [6,7,8]. To date, the compounds contributing to the distinctive “white onion” flavor of *B. roseoflavus* have not been comprehensively identified and analyzed. Furthermore, *B. roseoflavis* requires a specific ecological niche and complex symbiotic relationships for its growth, making artificial cultivation currently unfeasible. Therefore, sustainable harvesting practices are essential to avoid disrupting the local ecosystem.

Mushroom flavor results from a delicate interplay between volatile and non-volatile compounds, and its formation is influenced by multiple factors. For instance, the volatiles in fresh *Flammulina velutipes* are mainly composed of ketones and alcohols, with 3-octanone as the dominant component. Different packaging materials can alter its flavor during storage, while nano-packaging has been shown to slow the degradation of free amino acids, thereby preserving quality [9]. In *Agaricus bernardii,* the major volatile compounds include alcohols, ketones, and aldehydes—particularly 3-octanol, 3-octanone, 1-octen-3-ol, and benzyl alcohol. During storage, the contents of alcohols, aldehydes, hydrocarbons, and esters increase, whereas that of ketones declines [10]. Flavor profiles vary significantly among Boletus species with different genetic backgrounds. *Boletus edulis* is characterized by a strong roasted and buttery aroma, primarily attributed to 3-(methylthio)propanal and 2,6-dimethylpyrazine. *Boletus aereus* features a woody scent, with isovaleric acid, 2,6-dimethylpyrazine, phenylacetaldehyde, and (E)-2-octenal contributing notably to its flavor. *Boletus auripes* is distinguished by its intense floral and smoky notes, with key contributors including isovaleric acid, 3-ethylphenol, and 2,6-dimethylpyrazine [11]. It is widely recognized that abiotic factors associated with geographic origin significantly affect crop yield and quality [12,13,14,15], and such effects have also been observed in certain Basidiomycota mushrooms. For example, *Floccularia luteovirens* from Tibet contains the highest levels of unsaturated fatty acids and proteins, while samples from Qinghai exhibit the highest polyphenol content, and those from Sichuan are richest in polysaccharides [16]. In *Pleurotus* spp., samples from Indonesia show higher levels of gallic acid and p-hydroxybenzaldehyde, possibly due to tropical climate or cultivation conditions, whereas European samples are richer in coumaric acid and quercetin derivatives, likely influenced by strain or soil composition [17]. Moreover, mushroom aroma and taste are also affected by processing methods, cooking techniques, and harvest timing [18,19,20].

**Figure 1:**
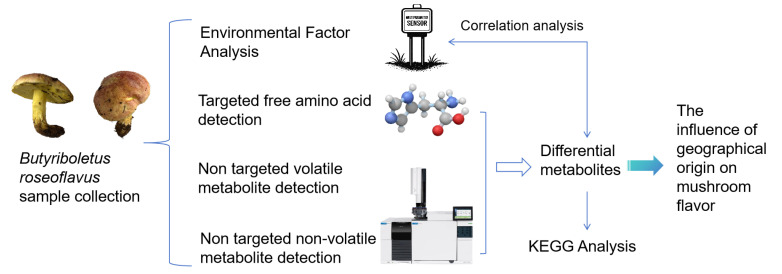
Schematic diagram of experimental design.

In summary, there is currently a lack of systematic identification of flavor compounds in *B. roseoflavis*, and no comprehensive studies have yet examined whether its flavor profile is significantly influenced by geographic origin and associated environmental factors. Therefore, this study employed integrated UPLC-MS/MS and HS-SPME-GC-MS analyses to characterize the volatile and non-volatile metabolite profiles of *B. roseoflavus* collected from different regions. Key flavor compounds were identified, and their potential associations with geographical and environmental variables were further explored (Figure 1). These findings provide a scientific foundation for the future development and utilization of *B. roseoflavus* as a high-value mushroom resource.

## 2. Materials and Methods

### 2.1. Sample Preparation

**Figure 2:**
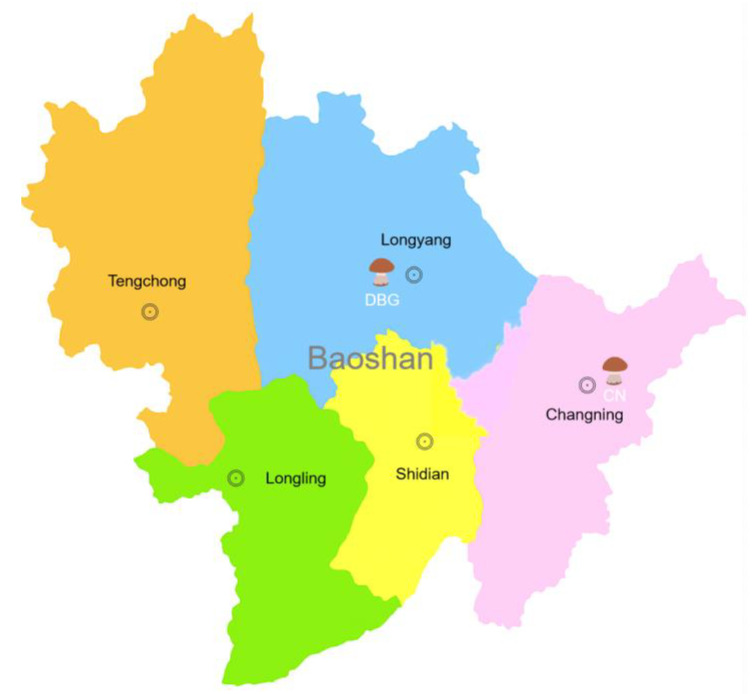
Schematic diagram of the collection location for *Butyroboletus roseoflavis*.

On 18 July 2024, *B. roseoflavis* samples were collected from two locations in Baoshan, Yunnan Province—Dabaogai (BDG; 25°12′ N, 99°12′ E) and Changning (CN; 24°86′ N, 99°67′ E) (Figure 2). Maturity was determined based on the color, shape, and size of the fruiting bodies. At each site, three samples of the same maturity stage were harvested and immediately preserved in liquid nitrogen for subsequent analysis.

### 2.2. Determination of Environmental Factors

Air temperature, air humidity, soil temperature, soil moisture, light intensity, conductivity, and CO_2_ concentration at the sampling sites were monitored using a multi-parameter sensor (Jiangsu Yunyuwu IoT Technology Co., Ltd., Nanjing, China). Measurements were taken three times daily, and daily averages were calculated. Altitude data were obtained in real-time using Ovi interactive map V10.2.6 software.

### 2.3. Molecular Identification

Genomic DNA was extracted from *B. roseoflavis* samples using a fungal DNA extraction kit (D2300, Beijing Solarbio Science & Technology Co., Ltd., Beijing, China). PCR amplification was performed using the universal primers ITS1 and ITS4 [21]. The 50 μL reaction mixture contained 45 μL of T6 Super PCR Mix Ver.2 (1.1×), 2 μL each of forward and reverse primers, and 1 μL of genomic DNA. The PCR program included an initial denaturation at 94 °C for 4 min, followed by 25 cycles of denaturation at 95 °C for 30 s, annealing at 39 °C for 30 s, and extension at 72 °C for 20 s, with a final extension at 72 °C for 10 min. PCR products were verified by gel electrophoresis, and the remaining products were purified and sequenced using a 3730XL automatic DNA sequencer (Applied Biosystems, Foster City, CA, USA). The assembled sequences were then analyzed using the NCBI BLAST tool 6.25 against the nucleotide database for species identification.

### 2.4. HS-SPME-GC–MS Analysis

#### 2.4.1. HS-SPME Extraction Conditions

The method was based on Li et al. [22] with slight modifications. A 500 mg ground *B. roseoflavus* sample was placed in a 20 mL headspace vial, to which 20 μL of 3-hexanone-2,2,4,4-d4 was added as the internal standard. The solid-phase microextraction (SPME) device used was SPME Arrow (CTC Analytics AG, Zwingen, Switzerland), with a 120 µm DVB/CWR/PDMS extraction fiber (Agilent, Austin, TX, USA). The extraction was performed at 60 °C under 5 min of shaking and 15 min of extraction. Desorption was carried out at 250 °C for 5 min.

#### 2.4.2. Chromatographic Conditions (Continued)

The column flow rate was 1.2 mL/min, and the injection port temperature was set to 250 °C. A non-split injection was used, and the temperature program for the column was as follows: 40 °C held for 3.5 min, then increased by 10 °C/min to 100 °C, followed by a 7 °C/min increase to 180 °C, and finally increased at 25 °C/min to 280 °C, where it was held for 5 min. The mass spectrometer was operated in electron ionization (EI) mode with an energy of 70 eV, and the ion source temperature was set to 280 °C.

#### 2.4.3. Volatile Metabolite Qualitative and Quantitative Analysis

A database was built using data on multiple species from the literature, standard reference compounds, and retention indices. This database contains retention times (RTs) and ions used for both quantification and qualification during SIM scanning. For each compound, one quantification ion and two or three qualification ions were selected. The ions were detected in each sample based on the peak sequence across continuous time intervals. Compounds were identified if their retention time matched the standard reference and the selected ions appeared in the mass spectrum after background subtraction [23]. Quantification ions were integrated and corrected to enhance accuracy. The relative content of volatile compounds was calculated using the internal standard semi-quantification method.

$$X_{i}=\frac{V_{s}\times C_{s}}{M}\times \frac{I_{i}}{I_{s}}\times 10^{-3}$$

X_i_ represents the concentration of compound i in the sample (μg/g); V_s_ is the volume (μL) of the internal standard added; C_s_ is the concentration of the internal standard (μg/mL); M is the mass (g) of the sample to be analyzed; I_s_ is the peak area corresponding to the internal standard; and I_i_ is the peak area of compound i in the sample to be analyzed.

### 2.5. Free Amino Acid Content Detection

Nineteen high-purity amino acid standards (>98%) were purchased from TCI Shanghai Chemical Industry Development Co., Ltd., Shanghai, China, including aspartic acid, glutamic acid, threonine, serine, glycine, alanine, valine, methionine, lysine, proline, isoleucine, leucine, tyrosine, phenylalanine, histidine, arginine, tryptophan, asparagine, and glutamine. Free amino acids (FAAs) were determined using an automatic amino acid analyzer (Sykam S 433D, Sykam GmbH, Eresing, Germany) according to the method outlined in GB 5009.124-2016 [24]. The column used was a sodium salt system with a flow rate of 0.2 mL/min, an injection volume of 0.05 mL, and dilution factors of 1× for standards and blanks, and 10× for samples. Absorbance was measured at 440 and 570 nm. Proline absorption was observed at 440 nm, and other amino acids were measured at 570 nm.

### 2.6. UPLC-MS/MS Analysis

#### 2.6.1. Sample Extraction

This was based on the method of Yang et al. [25], with slight modifications. A 50 mg ground *B. roseoflavus* sample was added to 1200 μL pre-chilled 70% methanol aqueous internal standard extraction solution. The mixture was vortexed five times using a VORTEX-5 vortex mixer (China) with 25 min intervals, followed by centrifugation at 11,000 rpm for 180 s. The supernatant was filtered using a 0.22 μm pore size membrane and analyzed by UPLC-MS/MS.

#### 2.6.2. Chromatographic Conditions

This was based on Yang et al. [25] with slight modifications. Non-volatile compounds were separated using a Waters chromatographic column (2.1 mm × 100 mm × 1.8 µm, Milford, MA, USA), installed on an LC-30A ultra-performance liquid chromatography system (Shimadzu, Kyoto, Japan). The mobile phase consisted of A: 0.1% formic acid in water and B: 0.1% formic acid in acetonitrile. The column temperature was set to 40 °C, the flow rate was 0.4 mL/min, and the injection volume was 4 μL.

#### 2.6.3. Mass Spectrometry Conditions

Mass spectrometry was performed on a TripleTOF 6600+ (SCIEX, Framingham, MA, USA) using electrospray ionization (ESI) in both positive (+) and negative (−) ion modes. The acquisition time was 10 min, with an ionization voltage of 5000 V and an ion source temperature of 550 °C. The MS1 collision energy was 10 V, with a mass range of 50–1000 Da and an acquisition time of 0.2 s. For MS2, the collision energy was set to 30 V, with a mass range of 25–1000 Da, and the acquisition time was 0.04 s.

#### 2.6.4. Metabolite Qualitative and Quantitative Analysis

The mass spectrometry data were processed using XCMS 4.5 software for peak extraction, baseline correction, and peak alignment. Peaks with a missing rate > 50% were excluded, and blank values were filled using KNN. The peak areas were corrected using the SVR method. Metabolites were identified using the NIST database, a self-built standard compound database, and a prediction library.

### 2.7. Taste Active Value (TAV) Analysis

TAV is a parameter determined by the ratio of the concentration (C) of a compound in food to its sensory detection threshold (T):

TAV = C/T

A TAV greater than 1 indicates that the compound is a characteristic flavor component. The higher the TAV, the greater its contribution to the overall flavor.

### 2.8. Relative Odor Active Value (rOAV) Analysis

The relative odor active value (rOAV) is calculated as the ratio of the relative content of each volatile compound to its odor threshold (OT). This method is commonly used to assess the contribution of individual volatile compounds to the overall aroma profile of a food product.

### 2.9. Statistical Analysis

Principal Component Analysis (PCA) and Orthogonal Partial Least Squares Discriminant Analysis (OPLS-DA) were performed using SIMCA 14.1 software. Bar graphs were created with Origin 2021 software. Data processing was conducted in Excel, and variance analysis was performed using SPSS 26.0 software. Each sample was analyzed in parallel three times, and the results are presented as mean ± standard deviation, with a significance level of 0.05.

## 3. Results and Discussion

### 3.1. Molecular Characteristics of B. roseoflavis

The collected samples were identified based on morphological features and rDNA ITS sequencing. The ITS sequences were submitted to GenBank under the accession numbers CN: PV570200 and BDG: PV570231. Sequence alignment with existing GenBank entries showed 99% similarity to *B. roseoflavis*. A phylogenetic tree was constructed using the neighbor-joining method (Supplementary Figure S1), revealing that the collected samples were closely related to *B. roseoflavus*, with a bootstrap value of 100. These results confirm that the wild mushroom samples were *B. roseoflavis* at the molecular level.

### 3.2. Environmental Factor Analysis

Environmental factors at the CN and DBG sampling sites were analyzed. As shown in Table S1, significant differences were found in environmental factors between the two locations. Specifically, DBG had significantly higher altitude and soil electrical conductivity than soilthan CN, while CN had significantly higher daily average soil temperature, daily average soil moisture, daily average air temperature, daily average air humidity, light intensity, and CO_2_ concentration compared to DBG.

### 3.3. Free Amino Acids (FAAs) Determination

**Figure 3:**
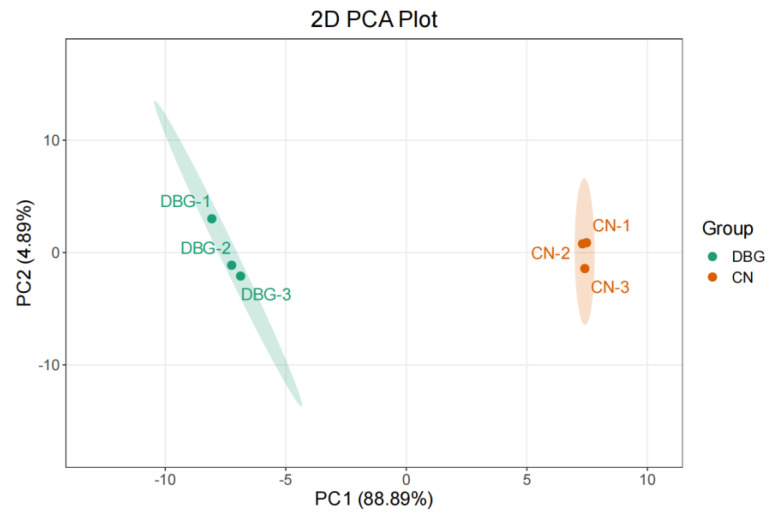
Free amino acid (FAA) principal component analysis plot.

Principal component analysis (PCA) was conducted on the content of 19 detected amino acids. The first and second principal components (PC1 and PC2) accounted for 81.77% and 9.72% of the total variance, respectively, with all samples clearly clustered according to their geographical origin (Figure 3). Table 1 lists the types, taste attributes, and contents of FAAs detected in *B. roseoflavus* from different regions. The total content of 15 free amino acids was significantly higher in the CN group compared to the DBG group. Regarding taste attributes, the CN group showed significantly higher levels of umami, sweet, bitter, and other amino acids compared to the DBG group. Regarding individual amino acid content, Glu and Gln were the most prominent in both DBG and CN. The content of Gly in the CN group was three times higher than in DBG, and Ser content was 2.6 times higher in the CN group. Table 2 presents the TAVs for the free amino acids. As shown in Table 2, 12 out of 19 free amino acids in the CN group had significantly higher TAVs than those in the DBG group. In the CN group, five free amino acids had TAVs greater than 1, which were identified as characteristic flavor components: Glu, Ala, Gly, Ser, and His. In the DBG group, four free amino acids had TAVs greater than 1, which were identified as characteristic flavor components: Glu, Ala, Arg, and His. This indicates that in CN, Glu is the major contributor to umami, Ala, Gly, and Ser contribute mainly to sweetness, and His is a major contributor to bitterness. In DBG, Glu contributes mainly to umami, Ala to sweetness, and Arg and His to bitterness. Based on these experimental results, we observed that the content of free amino acids in *B. roseoflavus* changed during growth, which influenced the umami, sweetness, and bitterness, leading to a more umami-sweet taste in CN compared to DBG. Guo et al. observed significant differences in amino acid content in ginkgo leaves growing in different locations [26], which is similar to what we observed in *B. roseoflavus* samples. We performed a correlation analysis between the free amino acid content of CN and DBG samples and their environmental conditions (Figure S2A). The results showed that the content of Glu, Asp, Gly, Ser, Arg, Ile, His, Leu, Trp, Lys, and Val was positively correlated with altitude and soil electrical conductivity, and negatively correlated with soil temperature, soil humidity, air temperature, air humidity, and light intensity. In contrast, Ala, Thr, Tyr, and Pro were negatively correlated with altitude and soil electrical conductivity, and positively correlated with soil temperature, soil humidity, air temperature, air humidity, and light intensity. Phe, Met, and Asn showed no significant correlation with environmental factors.

Free amino acids (FAAs) are essential flavor compounds in edible fungi [27] and have unique sensory characteristics, primarily responsible for the formation of components related to sweetness, bitterness, and umami (one of the five basic tastes) in edible fungi [28]. As precursors of volatile substances in biological systems, the conversion methods and pathways have been validated in various plants and plant organs [29,30,31]. For instance, Methionine (Met) acts as a precursor for a variety of sulfur and non-sulfur volatiles through the non-dependent pathway of the Yang cycle. In melon and strawberry fruits, methanethiol is further metabolized into volatile sulfur esters by acyl transferase-like enzymes. Phenylalanine (Phe) serves as a precursor for the biosynthesis of volatile phenylpropanoids, including eugenol and isoeugenol [32]. The composition, concentration, and degradation of FAAs can all impact the flavor quality of edible mushrooms [33]. It is well known that altitude has a compound effect on climate, resulting in regional variations in parameters such as diurnal temperature range, precipitation, and atmospheric pressure. Accordingly, it is speculated that the amino acid content in the samples is influenced by microclimatic conditions mediated by altitude. In addition, amino acid metabolism and regulation vary under different environmental conditions [34]. Lee et al. found that the amino acid content in tea leaves increased with reduced temperature, rainfall, and light duration [35]. Similarly, CN’s growing environment has higher humidity and lower temperature than DBG, leading to a significant increase in amino acid content. The underlying reason may be that environmental conditions directly affect the flow and distribution of carbon and nitrogen within the organism, thereby influencing amino acid accumulation [26].

### 3.4. Aroma Concentration and Composition Analysis of B. roseoflavus Samples

**Figure 4:**
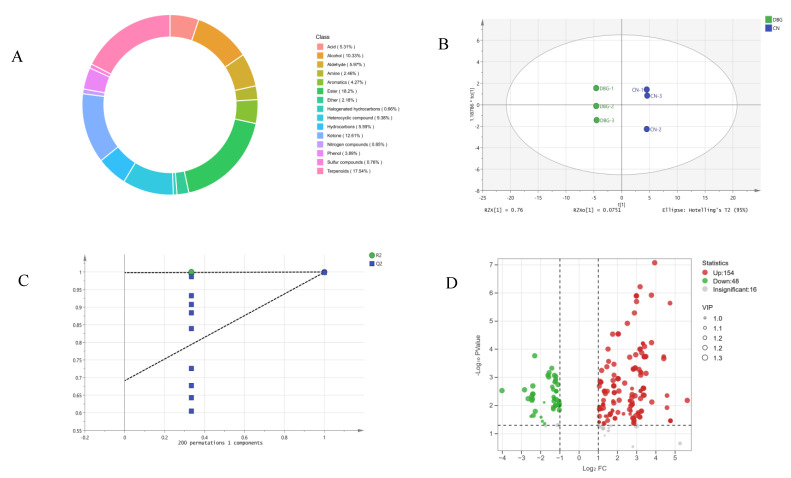
Comprehensive analysis of volatile metabolites: (**A**) volatile metabolite species; (**B**) scatter plot of scores from the orthogonal partial least squares discriminant analysis (OPLS-DA) model; (**C**) results of the 200-permutation test; and (**D**) volcano plot of differential metabolites.

The majority of precursors for the synthesis of volatile aromatic compounds in mushrooms are derived from various non-volatile substances, such as amino acids, fatty acids, carbohydrates, and terpenes [36]. Identifying the volatile aroma components and key flavor substances in mushrooms can provide insights into how metabolism and the growth environment impact the types and quantities of flavor compounds. This process also helps to identify internal factors that influence the production of these compounds [37]. Using the HS-SPME-GC-MS method, volatile compounds and their concentrations in the samples were detected and analyzed. A total of 1056 volatile compounds were identified (Figure 4A), including 17.54% terpenes, 18.2% lipids, 12.61% ketones, 10.33% alcohols, 9.38% heterocyclic compounds, 5.97% aldehydes, 5.59% hydrocarbons, 5.31% acids, 4.27% aromatics, 3.89% phenols, 2.46% amines, 2.18% ethers, and 2.27% other compounds. OPLS-DA analysis was conducted on the detected volatile compound content (Figure 4B). The R^2^X value (fitting index for independent variables) was 0.978, the R2Y value (fitting index for dependent variables) was 1, and the Q^2^ value (prediction index) was 0.999. R^2^ and Q^2^ values above 0.5 indicate good sample separation [38]. After 200 permutation tests (Figure 4C), the Q^2^ regression line intersected the vertical axis below zero, showing no overfitting and validating the model. This result clearly demonstrates the overall aroma differences between CN and DBG. Analysis of the relative content of the identified volatile compounds (Figure S3A) showed that DBG had a significantly higher relative total content than CN, with all categories except for ethers and other compounds being higher in DBG.

**Figure 5:**
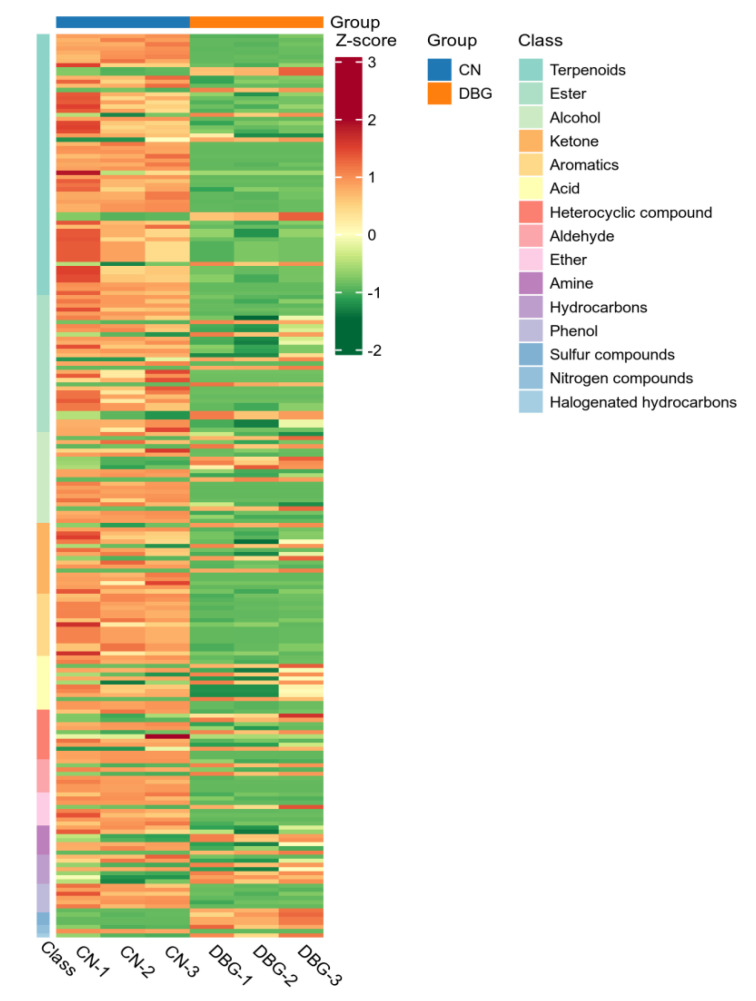
Heat map of volatile substance differential metabolites.

To further analyze the characteristic aroma substances of CN and DBG, differential metabolites were selected based on VIP > 1, fold change ≥ 2, and fold change ≤ 0.5. A total of 218 differential metabolites were screened, and the volcano plot (Figure 4D) showed that 154 metabolites were significantly upregulated and 48 metabolites significantly downregulated. The heat map of differential metabolite clustering (Figure 5) revealed that the three largest categories of volatile substances were terpenes, aldehydes, and alcohols. In fungi, terpenes are synthesized via the mevalonate (MVA) pathway, which uses acetyl-CoA as the starting substrate [36]. Aldehydes are an important class of volatile components in edible fungi, contributing green, fatty, almond, and waxy aromas [39]. Alcohols primarily provide a mushroom-like and green aroma, mainly produced via the fatty acid pathway. For example, octenol is formed during the oxidative decomposition of linoleic acid [40].

Relative odor activity value (rOAV) is a key tool used to assess the contribution of key aroma components to the overall mushroom aroma from both the concentration and threshold perspectives [41]. By combining rOAV, key aroma substances in DBG and CN were analyzed (Table 3). The results showed that 17 volatile compounds in CN had OAV > 1, including 4 aromatic hydrocarbons, 3 aldehydes, 3 esters, 2 ketones, 2 ethers, 1 phenol, 1 terpene, and 1 heterocyclic compound. In DBG, 10 volatile compounds had OAV > 1, including 2 ketones, 2 aldehydes, 2 esters, 1 aromatic hydrocarbon, 1 ether, 1 terpene, and 1 heterocyclic compound. These compounds play key roles in the aroma formation of *B. roseoflavus* from different regions. α-pinene, styrene, octanal, 1,3,5-trithiane, and 2,4-undecadienal had rOAV values > 10, presenting floral, fragrant oil, grassy, sulfurous, and buttery aromas, respectively, indicating that these are the major contributors to the aroma of *B. roseoflavus*. Notably, 13 out of the 17 key aroma substances had higher rOAV values in the CN group than in the DBG group, suggesting that *B. roseoflavus* from CN has a more intense aroma.

The Pearson correlation coefficient method was used to calculate the relationship between key aroma compounds and environmental factors (Figure S3B). The results showed that, except for ethyl acetate, 3-mercapto-2-pentanone, (+)-α-pinene, and octanal, key aroma compounds were significantly positively correlated with daily average air temperature, daily average air humidity, daily average soil temperature, daily average soil humidity, and daily light intensity. Research has demonstrated that the synthesis and accumulation of aromatic compounds in crops, including grains, tea leaves, flowers, and fruits, are affected by environmental factors such as temperature, humidity, and light conditions during their development. For instance, high temperatures and low rainfall before grape ripening lead to higher levels of phenolic compounds, contributing to weight variations in grape fruits, while cooler conditions favor an increase in terpene content in grapes [42]. Dry air or insufficient vapor pressure leads to the loss of aromatic compounds in grains, while humid air allows grains to accumulate stronger and more complex aromatic compounds [43]. This is likely due to abiotic stress enhancing the expression of aroma-synthesizing genes, leading to the accumulation of characteristic aroma compounds [44,45].

### 3.5. UPLC-MS/MS Analysis of B. roseoflavus Samples

**Figure 6:**
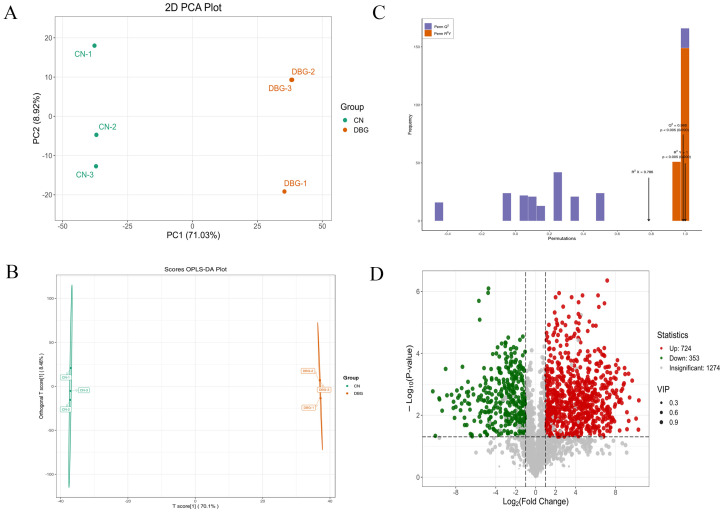
Comprehensive analysis of non-volatile metabolites: (**A**) non-volatile metabolite species; (**B**) scatterplot of scores from the orthogonal partial least squares discriminant analysis (OPLS-DA) model; (**C**) results of the 200-permutation test; and (**D**) volcano plots of differential metabolites.

PCA can be used to identify the overall metabolic differences and variability between sample groups. As shown in Figure 6A, the grouping ellipses of *B. roseoflavus* metabolites separated from the two regions were plotted. The contribution of PC1 was 71.03%, and PC2 was 8.92%. The clear trend in sample separation indicates significant differences between the samples. The total amount of non-volatile metabolites in DBG and CN was similar (Figure S3B). However, the levels of amino acids and their derivatives, organic acids, and alkaloids were higher in DBG than in CN, while the levels of benzene and its derivatives, lipids, nucleotides and derivatives, alcohols, amines, phenolic acids, heterocyclic compounds, flavonoids, and terpenes were lower in DBG than in CN. A total of 2351 metabolites were identified in *B. roseoflavus*, including 20.51% amino acids and their derivatives, 20.26% organic acids, 8.97% lipids, 8.85% benzene and its substituted derivatives, 7.18% nucleotides and derivatives, 3.72% alkaloids, 3.33% flavonoids, 3.21% phenolic acids, 2.95% terpenes, 2.18% total proteins, 1.92% alcohols and amines, 1.92% heterocyclic compounds, and 1.03% lignans and coumarins.

### 3.6. Differential Metabolite Screening

**Figure 7:**
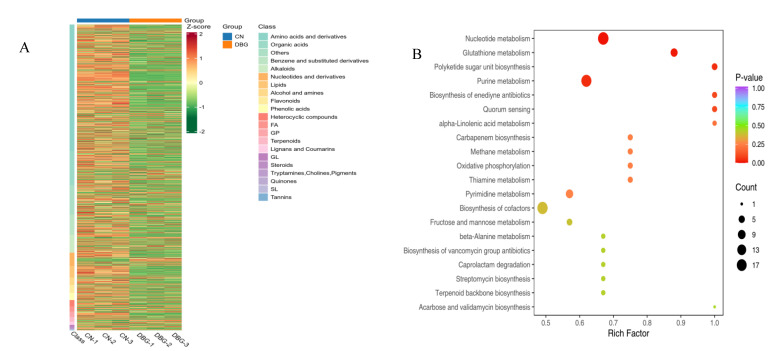
(**A**) Heatmap of differential metabolites of non-volatile substances; (**B**) Kyoto Encyclopedia of Genes and Genomes (KEGG) enrichment map of differential metabolites.

OPLS-DA was used to screen the metabolites corresponding to the differences between the two groups (Figure 6B,C). In our study, the R^2^X value was 0.786, the R^2^Y value was 1, and the Q^2^ value was 0.988, indicating that the OPLS-DA model is stable. Using VIP > 1, fold change ≥ 2, and fold change ≤ 0.5 as the selection criteria, 1226 non-volatile differential metabolites were identified between DBG and CN, with 724 metabolites upregulated and 353 metabolites downregulated (Figure 6D). To better visualize the relative content of these metabolites from different geographical sources of *B. roseoflavus*, a heatmap analysis was performed (Figure 7A). The higher the brightness of orange in the figure, the higher the abundance of metabolites, while the higher the brightness of green, the lower the abundance of metabolites. The difference in brightness between these colors helps us visually distinguish the differences between different groups more quickly. The results showed that the abundance of differential metabolites in CN was significantly higher than that in DBG.

### 3.7. KEGG Analysis of Differential Metabolites

The KEGG database can reveal the biological pathway information enriched by metabolites [46]. KEGG analysis revealed (Figure 7B) that the differential metabolites were involved in 82 KEGG pathways. The significantly different metabolic pathways included four pathways: glutathione metabolism, purine metabolism, nucleotide metabolism, and ketosugar unit biosynthesis. Redox reactions are fundamental to cellular energy conversion and are also important factors in regulating cellular responses to environmental stimuli. Glutathione is a crucial antioxidant, providing redox stability to cells and serving as an interface for metabolic reactions and signaling pathways, providing fuel for the growth and development of organisms. Additionally, it can regulate plant defenses and assist in the biosynthesis of sulfur-containing metabolites through various mechanisms [47]. In the glutathione metabolism pathway, seven metabolites were identified, and the level of gamma-glutamylcysteine in CN was significantly higher than in DBG. In purine metabolism, 18 metabolites were identified, with uric acid, deoxyguanosine 5′-monophosphate (dGMP), 2′-deoxyinosine, adenosine 5′-diphosphate (ADP), 2′-deoxyinosine triphosphate, and adenosine monophosphate levels significantly lower in CN than in DBG. In the nucleotide metabolism pathway, 20 metabolites were identified, and the levels of uridine, xanthosine, hypoxanthine, inosine, cytidine-5′-monophosphate, thymidine-5′-diphosphate, 2′-deoxyadenosine, guanine, guanosine, and 2′-deoxyadenosine-5′-monophosphate were significantly higher in CN than in DBG. In the ketosugar unit biosynthesis pathway, four metabolites were identified, and the level of dTDP-3-0-methyl-beta-L-rhamnose in CN was significantly higher than in DBG. Interestingly, the majority of these differential metabolites were concentrated in nucleotides and their derivatives. Research has shown that when plants face abiotic stress, some nucleotides and their derivatives act as signaling molecules, bridging the gap between perception and adaptation, coordinating cellular and physiological responses, and enabling better adaptation to environmental changes. Additionally, some nucleotides and their derivatives have recently been shown to play a central role in pathogen defense [48]. Therefore, we speculate that nucleotides and their derivatives play a critical role in the environmental adaptation and quality formation of *B. roseoflavus*.

## 4. Conclusions

*B. roseoflavis* from different geographic origins exhibits similar biological characteristics and cannot be easily distinguished by appearance. In this study, a total of 1056 volatile compounds were identified using HS-SPME-GC–MS, with terpenes, lipids, and ketones being the most abundant classes. Key contributors to the mushroom’s aroma included α-pinene, styrene, octanal, 1,3,5-trithiane, and 2,4-undecenal. Targeted metabolomics revealed significantly higher levels of free amino acids in the CN group than in the BDG group. TAV analysis indicated that CN had more taste-active compounds and higher TAVs. A total of 2351 metabolites were detected via UPLC-MS/MS, with differentially abundant compounds mainly concentrated in nucleotides and their derivatives, suggesting a critical role in environmental adaptation and flavor development. Correlation analysis showed that microclimatic changes mediated by altitude and soil electrical conductivity were the main factors driving taste differences, while temperature, humidity, light intensity, and CO_2_ concentration were the dominant factors affecting aroma divergence between CN and BDG. This study is the first to systematically uncover the metabolic mechanisms underlying geographic variation in the flavor of *B. roseoflavus*, clarifying the relationship between key flavor compounds and environmental factors. It also proposes a potential regulatory role for nucleotide-related metabolites in adaptation and flavor formation. These findings offer new insights into microbial responses to abiotic factors and lay a theoretical foundation for the future selection of high-quality germplasm resources.

## Figures and Tables

**Table 1:** Free amino acid (FAA) content (N = 3, average ± SEM).

Amino Acid	Taste Attribute	FAAs Content (mg/100 g)	
		CN	DBG
Glu	Umami	766.538 ± 12.28 b	525.525 ± 2.928 a
Asp	Umami	14.163 ± 0.336 b	16.154 ± 0.05 a
Umami FAAs		780.702 ± 12.475 b	541.679 ± 2.896 a
Ala	Sweet	224.178 ± 4.561 b	202.069 ± 1.594 a
Gly	Sweet	168.067 ± 5.437 b	56.984 ± 0.412 a
Thr	Sweet	81.371 ± 1.814 b	60.639 ± 0.578 a
Ser	Sweet	180.823 ± 7.561 b	69.907 ± 0.679 a
Sweet FAAs		654.439 ± 18.449 b	389.6 ± 2.237 a
Arg	Bitter	44.081 ± 1.168 b	58.113 ± 0.818 a
Ile	Bitter	46.034 ± 0.623 b	23.775 ± 0.267 a
His	Bitter	32.294 ± 0.854 b	25.718 ± 0.291 a
Leu	Bitter	32.327 ± 0.353 b	28.311 ± 0.514 a
Phe	Bitter	4.866 ± 0.085 a	4.805 ± 0.095 a
Tyr	Bitter	30.758 ± 0.691 b	35.116 ± 1.109 a
Trp	Bitter	7.902 ± 0.033 a	7.941 ± 0.237 a
Bitter FAAs		198.261 ± 3.649 b	183.778 ± 2.373 a
Lys	Bittersweet	21.443 ± 0.923 a	19.845 ± 0.095 a
Met	Bittersweet	6.985 ± 0.206 b	17.721 ± 0.139 a
Pro	Bittersweet	40.432 ± 0.757 b	34.826 ± 0.628 a
Val	Bittersweet	19.643 ± 0.418 b	16.418 ± 0.131 a
Bittersweet FAAs		88.503 ± 2.227 a	88.809 ± 0.989 a
Asn	Other	50.736 ± 0.758 b	44.752 ± 0.499 a
Gln	Other	652.776 ± 7.797 b	624.854 ± 2.544 a
Other FAAs		703.512 ± 8.525 b	669.605 ± 2.821 a
Total amount		2425.416 ± 44.729 b	1873.471 ± 7.596 a

Note: different lowercase letters in the same row represent significant differences between samples (*p* < 0.05).

**Table 2:** Taste active values (TAVs) of FAAs of *B. roseoflavus* (N = 3, average ± SEM).

Taste Compound	Taste Threshold (mg/100 g)	TAV	
		CN	DBG
Glu	30	25.551 ± 0.409 b	17.518 ± 0.098 a
Asp	53	0.267 ± 0.006 b	0.305 ± 0.001 a
Ala	60	25.819 ± 0.413 b	17.822 ± 0.097 a
Gly	130	3.736 ± 0.076 b	3.368 ± 0.027 a
Thr	260	1.293 ± 0.042 b	0.438 ± 0.003 a
Ser	150	0.313 ± 0.007 b	0.233 ± 0.002 a
Arg	50	1.206 ± 0.05 b	0.466 ± 0.005 a
Ile	90	6.548 ± 0.167 b	4.505 ± 0.029 a
His	20	0.882 ± 0.023 b	1.162 ± 0.016 a
Leu	190	0.512 ± 0.007 b	0.264 ± 0.003 a
Phe	90	1.615 ± 0.043 b	1.286 ± 0.015 a
Tyr	96.6	0.17 ± 0.002 b	0.149 ± 0.003 a
Trp	90	0.054 ± 0.001 a	0.053 ± 0.001 a
Lys	50	0.318 ± 0.007 b	0.364 ± 0.011 a
Met	30	0.088 ± 0 a	0.088 ± 0.003 a
Pro	300	3.638 ± 0.082 b	3.367 ± 0.028 a
Val	40	0.429 ± 0.018 a	0.397 ± 0.002 a
Asn	100	0.233 ± 0.007 b	0.591 ± 0.005 a
Gln	730	0.135 ± 0.003 b	0.116 ± 0.002 a

Note: different lowercase letters in the same row represent significant differences between samples (*p* < 0.05).

**Table 3:** Odor activity values (OAVs) of key volatile substances (N = 3, average ± SEM).

No.	Compounds	Threshold	OAVs	
		μg/g	DBG	CN
1	Propyl sulfide	0.003	1.302 ± 0.063 b	0.349 ± 0.031 a
2	Naphthalene, 2,7-dimethyl-	0.042	1.369 ± 0.088 b	0.128 ± 0.003 a
3	Naphthalene, 1,3-dimethyl-	0.042	1.369 ± 0.088 b	0.128 ± 0.003 a
4	Pentanoic acid, 4-methyl-, ethyl ester	0.006	1.517 ± 0.043 b	0.279 ± 0.001 a
5	3-Hexen-1-ol, acetate, (Z)-	0.031	3.198 ± 0.538 b	6.925 ± 0.336 a
6	Phenol, 2-methoxy-4-propyl-	0.0044	3.561 ± 0.041 b	0.375 ± 0.039 a
7	Butane, 1-(ethylthio)-	0.001	3.906 ± 0.19 b	1.047 ± 0.092 a
8	3-mercapto-2-pentanone	0.0007	4.412 ± 0.492 b	9.761 ± 0.23 a
9	5,9-Undecadien-2-one, 6,10-dimethyl-, (E)-	0.01	4.422 ± 0.435 b	2.016 ± 0.119 a
10	2,2,4-Trimethyl-1,3-pentanediol diisobutyrate	0.014	5.269 ± 0.324 b	1.465 ± 0.465 a
11	(1R)-2,6,6-Trimethylbicyclo [3.1.1]hept-2-ene	0.0053	5.664 ± 0.331 b	11.086 ± 0.675 a
12	Naphthalene, 2,6-dimethyl-	0.01	5.752 ± 0.37 b	0.536 ± 0.014 a
13	2,4-Undecadienal, (E,E)-	0.001	6.493 ± 0.165 b	0.684 ± 0.002 a
14	Styrene	0.0036	40.645 ± 0.737 b	11.353 ± 0.405 a
15	Octanal	0.0007	41.244 ± 4.394 b	120.834 ± 6.005 a
16	1,3,5-Trithiane	0.00004	359.051 ± 37.124 b	14.2 ± 8.097 a
17	2,4-Undecadienal	0.00001	649.305 ± 16.517 b	68.398 ± 0.17 a

Note: different lowercase letters in the same row represent significant differences between samples (*p* < 0.05).

## Data Availability

The data presented in this study are available on request from the corresponding author. (The data are not publicly available due to privacy or ethical restrictions.)

## Supplementary Materials

The following supporting information can be downloaded at: https://www.mdpi.com/article/10.3390/foods14101676/s1. Figure S1: Molecular phylogenetic tree of Butyriboletus roseoflavus based on ITS sequence analysis. Figure S2: Pearson correlation analysis (A) correlation analysis between free amino acids and environmental factors; (B) Correlation analysis of key volatile substances and environmental factors. Figure S3: Total metabolite content (A) concentration stacking diagram of volatile substances; (B) Stacking diagram of peak area of non-volatile substances. Table S1: Analysis of environmental factors of CN and DBG (N = 3, average ± SEM).
