# Supplementary material for: Identification of Characteristic Flavor Compounds and Quality Evaluation of Butyriboletus roseoflavus from Different Regions in Yunnan
Source: Foods. 2025 May 9;14(10):1676. doi: 10.3390/foods14101676 (PMC12111782; doi:10.3390/foods14101676)
Supplement: Supplementary file 1 [file foods-14-01676-s001.zip › foods-3604729-supplementary.pdf]

foods

MDPI

Supplementary Table S1

Analysis of environmental factors of CN and DBG (N = 3, average ± SEM).

Environmental factor	CN	DBG
Altitude (km)	1792.42 ± 27.08 b	2405.35 ± 45.37a
Air temperature (℃)	20.5 ± 0.19 b	18.57 ± 0.13a
Air humidity (% RH)	92.91 ± 0.44 b	88.87 ± 0.57a
Soil temperature (℃)	20.92 ± 0.08 b	18.21 ± 0.03a
Soil moisture (% RH)	26.58 ± 0.75 b	20.01 ± 0.21a
Illuminance (Lux)	2849.95 ± 157.6 b	1355.06 ± 141.15a
Conductivity (μS/cm)	48.5 ± 0.02 b	48.99 ± 0.02a
Carbon dioxide concentration (ppm)	639.86 ± 0.38 b	533.32 ± 1.7a

Note: different lowercase letters in the same row represent significant differences between samples ($p < 0.05$).

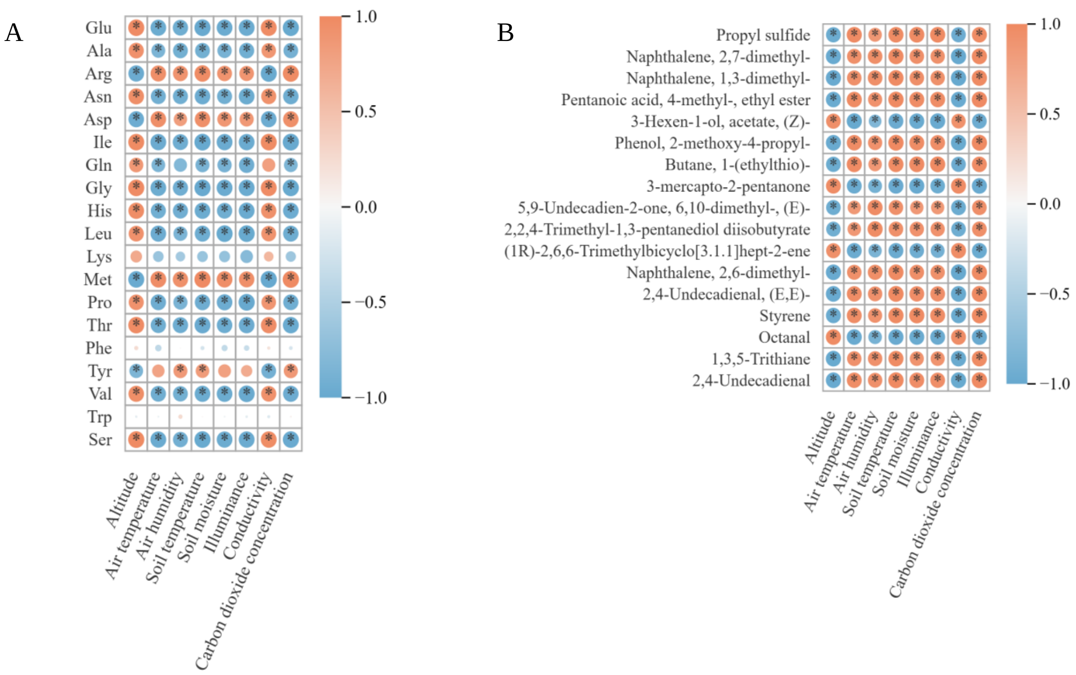

Supplementary Figure 1. Molecular phylogenetic tree of *Butyriboletus roseoflavus* based on ITS sequence analysis.

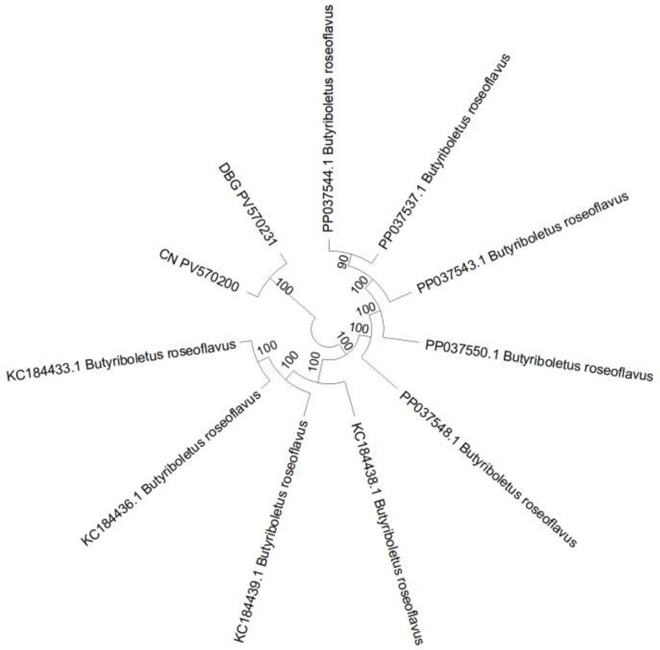

Supplementary Figure 2. Pearson correlation analysis (A) correlation analysis between free amino acids and environmental factors; (B) Correlation analysis of key volatile substances and environmental factors.

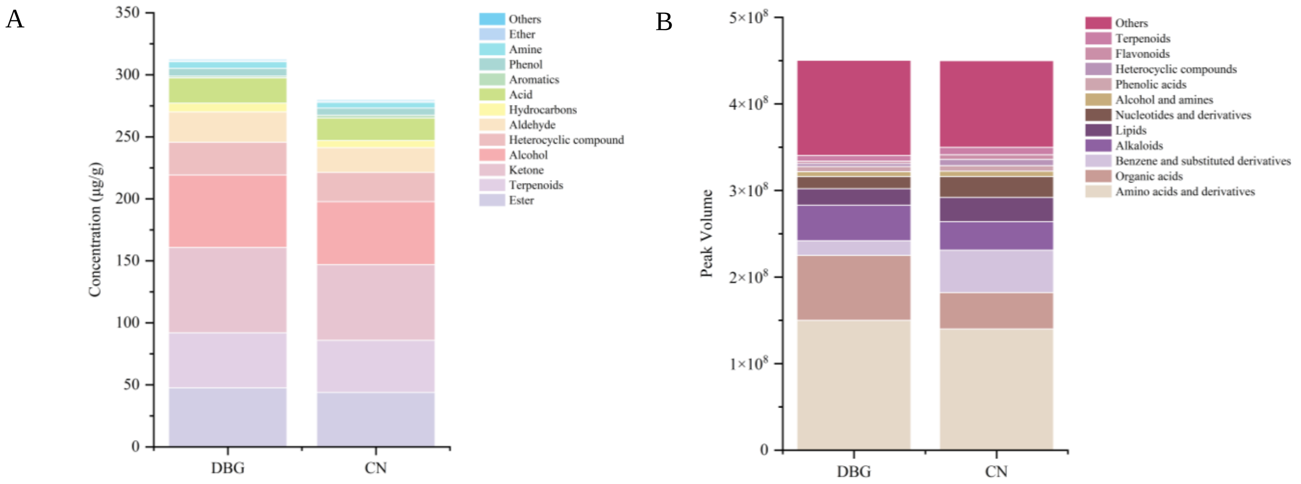

Supplementary Figure 3. Total metabolite content (A) concentration stacking diagram of volatile substances; (B) Stacking diagram of peak area of non-volatile substances.
